# Identification of Protozoa in Dairy Lagoon Wastewater that Consume *Escherichia coli* O157:H7 Preferentially

**DOI:** 10.1371/journal.pone.0015671

**Published:** 2010-12-20

**Authors:** Subbarao V. Ravva, Chester Z. Sarreal, Robert E. Mandrell

**Affiliations:** Produce Safety and Microbiology Research Unit, United States Department of Agriculture, Agriculture Research Service, Western Regional Research Center, Albany, California, United States of America; University of Osnabrueck, Germany

## Abstract

*Escherichia coli* O157:H7 (EcO157), an agent of life threatening hemolytic-uremic syndrome, resides in ruminants and is released in feces at numbers as high as 10 million cells/gram. EcO157 could survive in manure for as long as 21 months, but we observed a 90% decrease in cells of an outbreak strain of EcO157 within half a day in wastewater from dairy lagoons. Although chemical, environmental and biological factors may be responsible for this decrease, we observed an 11-fold increase in native protozoa when wastewater was re-inoculated with 2×10^7^ cells of EcO157/mL. These protozoa engulfed the green fluorescent protein labeled EcO157 within 2 hours after inoculation, but expelled vacuoles filled with live EcO157 cells within 3 days into surrounding wastewater, whereas other protozoa retained the EcO157-filled vacuoles for 7 days. EcO157 was not detected by confocal microscopy either inside or outside protozoa after 7 days. Mixed cultures of protozoa enriched from wastewater consumed EcO157 preferentially as compared to native aerobic bacteria, but failed to eliminate them when EcO157 cells declined to 10^4^/mL. We isolated three protozoa from mixed cultures and typed them by 18S sequencing as *Vorticella microstoma*, *Platyophyra* sp. and *Colpoda aspera*. While all three protozoa internalized EcO157, only *Platyophyra* and *Colpoda* acted as predators. Similar to mixed cultures, these protozoa failed to eliminate EcO157 from PBS containing no other supplemental nutrients or prey. However, spiking PBS with cereal grass medium as nutrients induced predation of EcO157 by *Platyophyra* sp. after 3 days or enhanced predation by *Colpoda* after 5 days. Therefore, attempts to enrich protozoa to decrease EcO157 from dairy lagoons, may correspond to an increase in protozoa similar to *Vorticella* and possibly facilitate transport of bacterial pathogens to food crops grown in proximity.

## Introduction

Produce was linked to 23% of 117,136 foodborne illnesses associated with outbreaks occurred during 1998 and 2007 in the United States [1]. *Escherichia coli* O157:H7 (EcO157) infections were responsible for 5% of the 684 produce associated outbreaks during the same period and 19% of all EcO157 associated outbreaks were due to consumption of contaminated produce [1]. In addition, major outbreaks associated with produce occurring in the US and in other parts of the world indicate that contamination has occurred in many cases in the field (pre-harvest), so it is critical to identify sources of pathogens in the environment and interventions for minimizing them [2]. Since pathogen contamination on ‘ready to eat’ produce cannot be removed easily [3], prevention of pre-harvest contamination is critical. Pre-harvest contamination emphasizes the importance of identifying animal reservoirs and the biological and environmental factors that regulate the proliferation of pathogens during their transport from ranches or dairies or wildlife to produce grown in proximity to these locations.

Although highly prevalent in some cattle herds in the US [4], the shedding of EcO157 is transient but elevated during summer and fall months [5]. Even if fecal shedding of pathogen is heavy during summer months, it has been reported in some studies that the pathogen disappears rapidly as the temperature increases during summer [6], [7]. However, we reported previously the lack of detection of the EcO157 in manure and wastewater collected from dairies in the central valley of California between 2001 to 2003 [6]. In contrast, EcO157 was isolated from watersheds, cattle herds, and wild pigs on the central coast of California throughout spring and summer months of 2004–2006 [8]. These results indicate a dynamic incidence that depends on spatial and temporal factors and possibly agricultural practices.

Pathogenic EcO157 often escapes detection because of its low numbers or, possibly, because it is concealed in alternate hosts such as flies, birds, dogs, cats, rodents, horses [9] and protozoa [10], [11]. Protozoa have gained notoriety as the ‘Trojan horses’ [12] of bacterial pathogens. *Salmonella enterica* serotype Typhimurium phage type DT104 [13], *Legionella pneumophila* [14] and *Mycobacterium avium* [15] passaged through protozoa have been reported to become hypervirulent; and coliforms (including non-pathogenic *E. coli*) and other pathogenic bacteria demonstrated increased resistance to chlorine disinfection [16]. It has been shown recently that Shiga toxin encoding prophage augmented the fitness of EcO157 in the presence of grazing protozoa, *Tetrahymena pyriformis* [17], thus providing a potential environmental selection pressure for bacteria to retain prophage encoding Shiga toxin. Although, EcO157 was known to survive and proliferate in co-culture with protozoa [12], [18], a predatory interaction could not be established between rumen ciliate protozoa and Shiga-toxigenic *E. coli* [19].

Protozoa constitute half of the microbial biomass in rumen and are essential in providing nutrients to the host through a symbiotic relationship with other rumen inhabitants [20]. Ruminants are primary reservoirs of EcO157 and may sometimes shed as many as 10^7^ per gram of feces [21], but there are no reports of evidence for the consumption of EcO157 by rumen protozoa. However, protozoan predation was linked indirectly to the decrease of EcO157 in well waters [22] and wastewater from a dairy [23]. Direct evidence for predation was reported recently for enteropathogenic *E. coli* in co-culture with *Acanthamoeba polyphaga* [24]. However, none of these studies have addressed EcO157 and protozoa in dairy wastewater even though both predator and prey in feces end up in lagoons. Since, EcO157 survived for 21 months in manure [25], but declined rapidly in wastewater from lagoons [6], we monitored protozoa in wastewater from dairy lagoons to determine if they are responsible for the decrease in EcO157. In addition, protozoa were isolated, identified by 18S rRNA sequences and evaluated further for their ability to consume and internalize EcO157. We used an outbreak strain of EcO157 marked with green fluorescent protein (GFP) reporter to confirm internalization of EcO157 in natural protozoa and to monitor the influence of nutrients and resident bacteria on protozoan predation in wastewater.

## Materials and Methods

### Enumeration of protozoa, EcO157, aerobic and coliform bacteria

Protozoa from 10-fold serial dilutions of wastewater samples were determined using the most-probable number (MPN) method [26]. Triplicate 50-µL portions of each dilution in optically clear 96-well polystyrene plates (Corning, Inc., Corning, NY) were monitored for protozoa using 40 to 200× magnification of an inverted microscope (Leica DM IL, Leica Microsystems, Wetzlar, Germany). Minimum detectability by the MPN method was 3.6 protozoan cells per 50 µL samples.

The populations of rifampin and nalidixic acid resistant EcO157 were monitored by plating 100-µL portions of serial dilutions of wastewater in 0.01M PBS on LB agar supplemented with 100 µg/mL rifampin, and 50 µg/mL each of nalidixic acid and cycloheximide (Sigma-Aldrich, St. Louis, MO). Cycloheximide was used to control fungal growth on enumeration media. The populations of GFP-EcO157 strain MM123 were monitored by plating the serial dilutions on LB agar supplemented with 100 µg/mL rifampin, and 50 µg/mL each of cycloheximide and kanamycin. The plated media were incubated overnight at 37°C and counted, except that the fluorescent colonies of MM123 were counted on a UV Transilluminator (Fotodyne, Hartland, WI).

One-mL portions of serial dilutions of wastewater were plated on Petrifilm count plates (3M Microbiology Products, St Paul, MN) to enumerate aerobic and coliform bacteria [6].

### Protozoan response in wastewater to repeat inoculation of EcO157

Native protozoan populations were monitored in wastewater supplemented with or without EcO157 strain MM123 as alternate prey. MM123 is a spontaneous rifampin-resistant (100 µg/mL) mutant of GFP-labeled strain RM2315 (original designation SEA13B88; provided by S. Wegant, FDA-Bothell, WA), which has been described previously [6], [27]. The wild type strain was the cause of a major outbreak associated with non-pasteurized apple juice [28]. Wastewater was collected from an aerated manure lagoon from a medium-sized dairy (ca. 800 milking head) in central California (Oakdale, CA) and acclimated overnight at room temperature prior to use [23]. Wastewater supernatant, similar to dairy wastewater with solids removed and reused to wash free-stall lanes on the diary, was used to accommodate enumeration of motile protozoa by the MPN method. Bottom feeding amoebae settling with manure solids were avoided. Tests were conducted in triplicate 250-mL Erlenmeyer flasks containing 100 mL of wastewater samples inoculated with two different levels of MM123: 1.1±0.2×10^5^ and 8.6±0.7×10^7^ CFU/mL. Overnight growth of EcO157 in LB broth supplemented with 100 µg/mL of rifampin and 50 µg/mL of kanamycin was centrifuged and resuspended in 0.01M PBS was used as a food source for protozoa. The flasks were incubated at 25°C on a gyratory shaker operated at low speed (50 rpm) to simulate circulating aeration in the lagoons, and samples were monitored at various intervals for GFP-EcO157, protozoa and aerobic bacteria. The microcosms containing the high inoculum were re-inoculated with 1.7±0.2×10^7^ CFU/mL after 7 days.

### Confirmation by confocal microscopy of protozoan feeding of EcO157

Wastewater samples from the experiment described above were examined at various intervals to determine the diversity of native protozoa that engulf GFP-EcO157. Five-hundred-microliter samples of wastewater containing 0.3% Polyox (*c.* 4,000,000 mol wt, Sigma-Aldrich) were stained with 40 mM propidium iodide and 10 mM SYTO 59 (Invitrogen, Carlsbad, CA) for 1 h to stain dead and live bacteria, respectively, then visualized by scanning on a Leica TCS-NT confocal microscope. Polyox enabled visualization by slowing down the protozoan movement. The internalization of EcO157 was determined by scanning through the total depth of protozoa as 5 to 10 µm optical sections. The sections were compiled using the Leica TCS NT Software and the fluorescence by GFP, propidium iodide, and SYTO 59 were assigned green, red and blue colors, respectively, for compiled images.

The diversity of protozoa in 10-fold concentrated wastewater samples was also determined by phase contrast microscopy using a Leica DMR microscope after staining the samples for 1 h with 1 µL each of neutral red (100 µg/mL; Sigma-Aldrich) and acridine orange (1 µg/mL; Invitrogen), and diluting samples with an equal portion of glycerol to decrease protozoan motility.

### Consumption of EcO157 by a mixed culture of natural protozoa from wastewater

Consumption of EcO157 strain MM123 by protozoa was determined using natural protozoa enriched from wastewater. One liter of wastewater was filtered through a 250 µm sieve, centrifuged (300×g, 10 min) and the pellet containing protozoa was transferred in 10 mL of 0.01M PBS to 250 mL of Sonneborn medium (Solution 1 of ATCC medium 802, https://www.atcc.org/Attachments/4018.pdf, Accessed 6 October 2010) supplemented with 5% of 0.2-µm-filter-sterilized wastewater (hereafter referred to as cereal grass-wastewater medium [CGWM]). A 7-day old growth of protozoa from CGWM was concentrated at 300×g and suspended in 0.01M PBS as the inoculum to test predation. Tests were conducted in triplicate 250-mL Erlenmeyer flasks containing 50-mL of CGWM inoculated with 2×10^7^ cells of EcO157 and 8×10^3^ cells of enriched protozoa per mL (2,500 bacteria/protozoa). CGWM inoculated with only MM123 was used as a no-predation control. The populations of GFP-MM123, protozoa and aerobic bacteria were monitored at various intervals.

### Isolation, purification and 18S rRNA characterization of protozoan monocultures

Protozoan enrichments described above were transferred sequentially 3 times at 7-day intervals to 250-mL portions of CGWM and incubated at 25°C on a gyratory shaker operated at 50 rpm. After the third transfer, 100-µL portions of ten-fold serial dilutions of the growth was transferred to optically clear 96-well polystyrene plates (Corning) with each well containing 100-µL cereal grass medium without the supplemental wastewater (CGM) and incubated at 25°C. Purity of protozoan cultures in 96-well plates was checked microscopically at periodic intervals and purified further by transferring 10-fold serial dilutions at 7-day intervals to fresh 100-µL of CGM until they were confirmed pure by microscopy.

Protozoa were grown in 100 mL-portions of CGM, concentrated by centrifugation at 300×g for 10 min and the cell pellet was used to extract DNA used for the amplification and sequencing of 18S rRNA as described in Method S1.

### Consumption of EcO157 by monocultures of protozoa from wastewater

Consumption of EcO157 strain MM123 was determined using three cultures of protozoa isolated from wastewater. Protozoan growth from 250-mL CGM supplemented with 50 µg/mL of tetracycline was centrifuged (300×g, 10 min), the pellet was washed twice in 100 mL of 0.01M PBS to remove bacterial carry-over, and re-suspended in 100 mL PBS as the inoculum to test predation. Tests were conducted in triplicate 250-mL Erlenmeyer flasks containing 50 mL of PBS inoculated with 1.1×10^7^ cells/mL of EcO157 and 1.1×10^3^ cells/mL of *Vorticella microstoma* or 1.5×10^2^ cells/mL of *Platyophyra* sp or 2.1×10^3^ cells/mL of *Colpoda aspera*. These treatments provide ∼10,000, 70,000 and 5,000 cells of EcO157 as food for each one of the *Vorticella*, *Platyophyra* and *Colpoda* cells, respectively. The survival of EcO157 in the absence of predation was monitored in PBS inoculated with only EcO157. As protozoa appeared to slow down in PBS, the treatments were supplemented with 5% CGM on day 3 for *Vorticella* and *Platyophyra* and on day 6 for *Colpoda*. The populations of GFP-EcO157, protozoa and aerobic bacteria were monitored at various intervals.

### Supplementation of wastewater to increase EcO157 and native bacteria in wastewater and stimulate predation

In an attempt to increase the numbers of native protozoa that consume EcO157, the wastewater was supplemented with or without 10% (w/v) LB broth and inoculated with two different concentrations (7.3±0.7×10^6^ and 6.6±0.9×10^8^ CFU/mL) of EcO157 strain MM149 as a supplementary food source (850 and 77,000 cells of EcO157 per protozoan, respectively). MM149 was isolated from dairy manure in northwestern Oregon and was selected for rifampin (110 µg/mL) and nalidixic acid (50 µg/mL) resistance to aid in discriminating the colonies from the bacteria in wastewater during enumerations [23]. Overnight growth of MM149 in LB broth supplemented with 100 µg/mL of rifampin and 50 µg/mL of nalidixic acid, centrifuged and resuspended in 0.01M PBS was used as food source for protozoa. The treatments were in triplicate and the populations of EcO157, protozoa, aerobic and coliform bacteria were monitored for 8 days.

### Data analysis

Repeated-measures 2-way ANOVA (Prism 4.0; GraphPad Software, Inc., San Diego, CA) was used to compare differences in growth of protozoa, EcO157, coliforms, and aerobic bacteria in wastewaters. All comparisons were in triplicate. Bonferroni post hoc test was used to determine the earliest interval at which the decline of EcO157 or growth of protozoa differed significantly (*P*<0.05) between various treatments.

## Results

### Protozoa in wastewater are stimulated by sequential inoculation of EcO157

**Figure 1:**
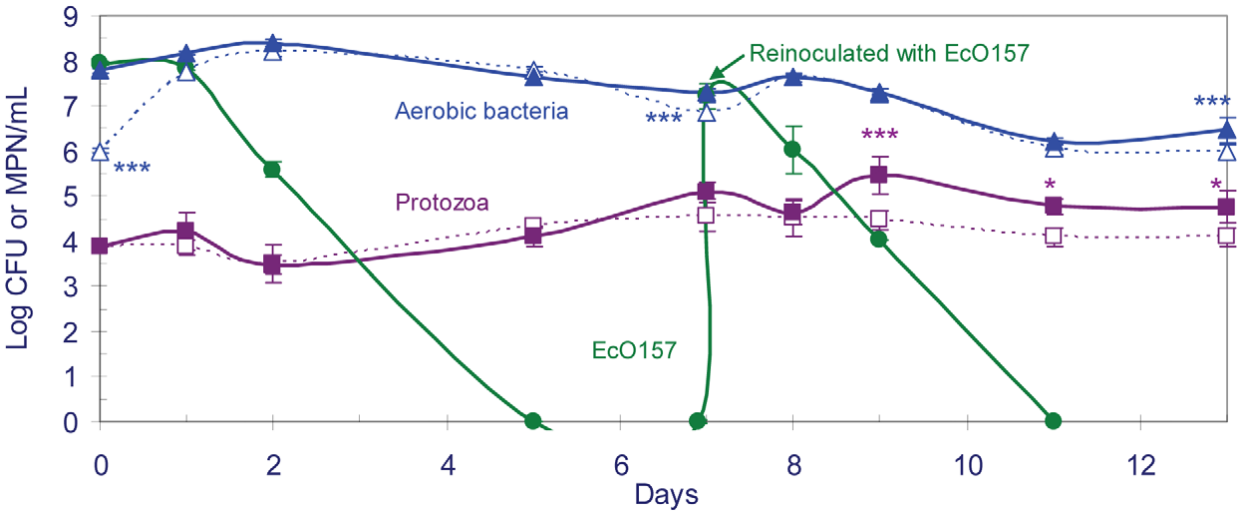
Increase in native protozoan numbers due to a repeat inoculation of GFP-EcO157 in wastewater. Populations of protozoa (▪, □; violet), aerobic bacteria (▴, ▵; blue) and EcO157 strain MM123 (•; green) were monitored in wastewater inoculated with (solid lines) or without (broken lines) EcO157. Wastewater was re-inoculated soon after the first batch of 9×10^7^ CFU of EcO157/mL declined to undetectable levels (7 days). Repeated-measures two-way ANOVA indicated significant increases in protozoa (*P* = 0.0014) with EcO157 treatment. Source of variation with time was *P*<0.0001. Bonferroni post hoc tests: *** = *P*<0.001 and * = *P*<0.05.

These studies and microscopy experiments were facilitated with a GFP-labeled strain of EcO157 (MM123). After an initial 1-day lag, EcO157 populations declined rapidly with a decrease of 90% populations within 0.5 day for both inoculum levels, and also after the second re-inoculation to samples treated with the higher inoculum of EcO157 (Figure 1). MPN counts indicated that each protozoan was provided initially with 14 or 11,200 cells of EcO157 for grazing and we anticipated that the higher level would support protozoan growth. Although no increase in protozoa was observed after the first treatment of 9×10^7^ cells of EcO157/mL (Figure 1), a repeat inoculation at 7 days resulted in an 11-fold increase in protozoa at 9 days (an increase from 3.2×10^4^ to 3.5×10^5^ protozoa/mL; *P*<0.001) after the start of the experiment. EcO157 cells decreased from 2.1×10^7^ to 1.5×10^4^ (a 99.9% decrease) during the same period and resulted in a decline in ratio of EcO157 to protozoa from 122∶1 to 0.04∶1. The spike in aerobic bacterial counts (*P*<0.001) at zero and 7 days was a result of enumerating the inocula as aerobic bacteria. At the low inoculum level of 10^5^ CFU EcO157/mL, the populations of protozoa and aerobic bacteria remained essentially the same as in uninoculated wastewater (not shown).

### Internalization of EcO157 in wastewater protozoa

**Figure 2:**
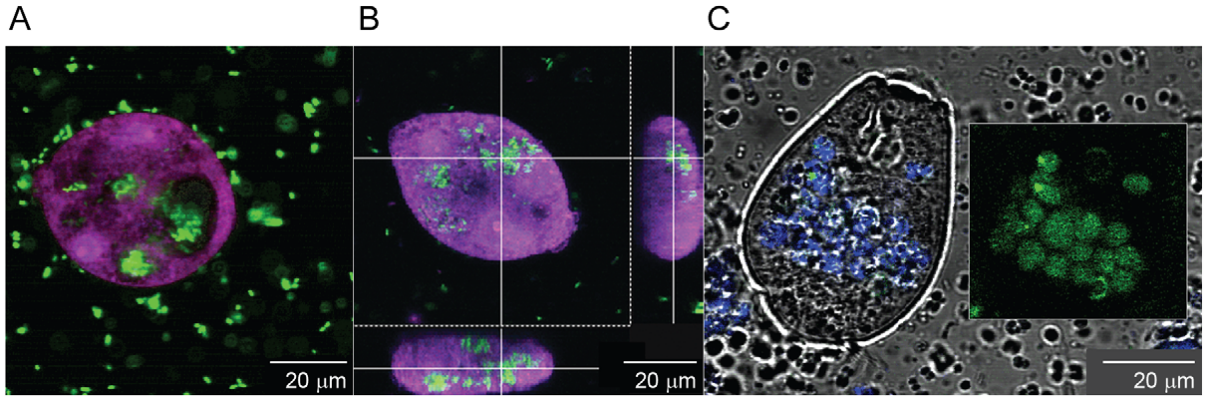
Internalization of GFP-EcO157 by protozoa in wastewater. Confocal images were taken within 2 h (A), 3 days (B) and 7 days (C) after inoculation of wastewater with 9×10^7^ CFU of GFP-EcO157 strain MM123/mL. Internalization of GFP-EcO157 cells was confirmed with compiled optical sections of protozoa (B). Blue coloration of the transmitted image (C) show live bacterial cells stained with Syto 59. The inset of GFP-EcO157 filled food vacuoles imply that live EcO157 cells were present in protozoa even after 7 days. EcO157 cells were not detected in the water surrounding protozoa.

GFP-EcO157 (MM123) cells engulfed in a variety of predatory protozoa were observed by confocal laser scanning microscopy of wastewater samples. GFP-EcO157 cells were sparsely distributed in the surrounding wastewater at the lower inoculum level (10^5^ CFU/mL) and could not be detected inside protozoa (Figure S1). However, at the higher inoculum level, the predominant protozoa that consumed EcO157 measured between 10 to 100 µm in diameter and appear to be ciliated. We captured numerous images of motile verticellid-like organisms (Figure 2A to 2C) that ingested the GFP-labeled cells within 2 h (Figure 2A) after inoculation and remained internalized even after 7 days (Figure 2C). EcO157 cells abundant before day 3 (Figure 2B) appear to be nearly absent from wastewater after 4 days (Figure S2), but remained clustered inside some protozoa at day 7 (Figure 2C). Live bacteria in vesicles stained with Syto-59 (colored pseudo blue) were GFP-EcO157 (inset, Figure 2C). Ten samples evaluated at 7 days confirmed the absence of GFP-labeled cells in the surrounding wastewater.

**Figure 3:**
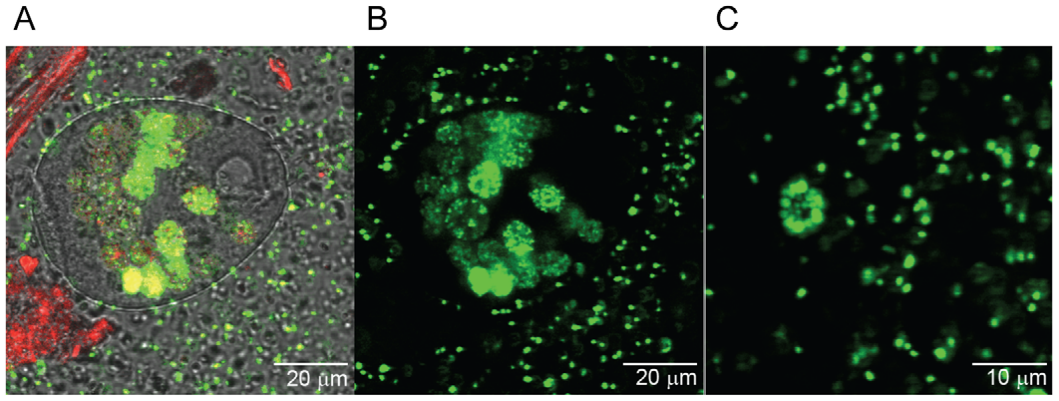
A native protozoan with food vacuoles packed with GFP-EcO157 and an expelled vacuole containing EcO157. Images were taken 3 days after inoculating the wastewater with GFP-EcO157 strain MM123. Confocal image (A) obtained with transmitted light in one of the three channels to gain definition of protozoan morphology. The yellowing of some food vacuoles (A) represents a high density number of GFP-EcO157 altering the fluorescence detection. This was confirmed by viewing the GFP-channel (B) depicting food vacuoles packed with bright green colored live EcO157 cells. Panel C shows an optical section of the expelled food vacuole with live EcO157. Very few dead cells (colored red) were observed (A).

Internalization of EcO157 was confirmed from compiled optical sections illustrating that GFP-EcO157 cells were inside a protozoan (Figure 2B). However, it should be noted that GFP-EcO157 cells were observed also attached to the surfaces of protozoa (Figure S3). We also observed the release of a vesicle containing GFP-labeled cells (Figure 3C) from a vorticellid (Figure 3A) packed with EcO157 cells within 3 days after grazing. Cross-sectional confocal laser scans of protozoa revealed that the food vacuoles were densely packed with GFP (Figure 3A and 3B, GFP-panel). Although we observed an occasional flagellate that consumed EcO157 (Figure S2), not every type of protozoa in wastewater consumed EcO157, even at the higher inoculum level. Some appeared refractile and the cyst-like structures did not consume any EcO157 throughout the incubations. We did not find any amoebae by either confocal or phase-contrast microscopy.

### Preferential consumption of EcO157 by protozoa enriched from wastewater

Protozoa from wastewater were enriched in nutrient media and tested for their ability to consume GFP-EcO157 strain MM123 relative to other aerobic bacteria enriched from wastewater. Potential inhibitory compounds from wastewater [23] were minimized by supplementing CGM with only 5% of filter-sterilized wastewater. However, wastewater supplementation was required to prevent the rapid die-off of enriched protozoa from CGWM.

**Figure 4:**
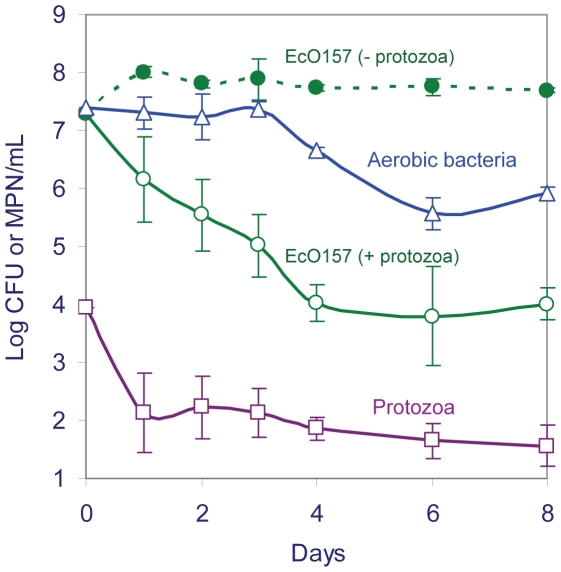
Enhanced consumption of EcO157 by protozoa enriched from wastewater. The decline in numbers of GFP-EcO157 strain MM123 (○, green) in cereal grass medium supplemented with 0.2-µm-filtered wastewater inoculated with a mixed culture of protozoa was compared with the fate of EcO157 (•, green) in the medium without the protozoa. Total protozoa (□, violet) and aerobic bacteria (▵, blue) were monitored.

The number of EcO157 declined rapidly during the first 4 days in the presence of protozoa (Figure 4), whereas they grew 5-fold on day 1 (an increase from 2×10^7^ to 1×10^8^ cells/mL) in the absence of protozoa and maintained at an elevated level of 5×10^7^ CFU/mL after 4 days. The number of aerobic bacteria remained stable during the first 3 days (3×10^7^ at day 3), whereas the cells of EcO157 were consumed preferentially (1×10^5^ at day 3). After 3 days, the protozoa switched to grazing aerobic bacteria after 99.6% of EcO157 cells were consumed. The ratio of aerobic bacteria to protozoa on the 3^rd^ day was 189,000∶1, whereas the EcO157 cells to protozoan ratio declined to 630∶1. However, protozoan numbers also declined within a day, but maintained at 79±3 cells/mL after the initial decline.

### Predation of EcO157 by monocultures of protozoa isolated from wastewater

Protozoan diversity was observed to decrease from 8 to 10 morphological types to 5 types during the transition from natural wastewater to CGM supplemented with 5% filtered wastewater. Purification by serial dilutions and culturing in CGM resulted in the isolation of three ciliates characterized by 18S rRNA sequencing as *Vorticella microstoma* (GenBank accession # DQ868347), *Platyophyra* sp. (EU039905) and *Colpoda aspera* (EU039892). All the sequences obtained from 5 clones selected for each protozoan matched 100% with the GenBank sequences.

**Figure 5:**
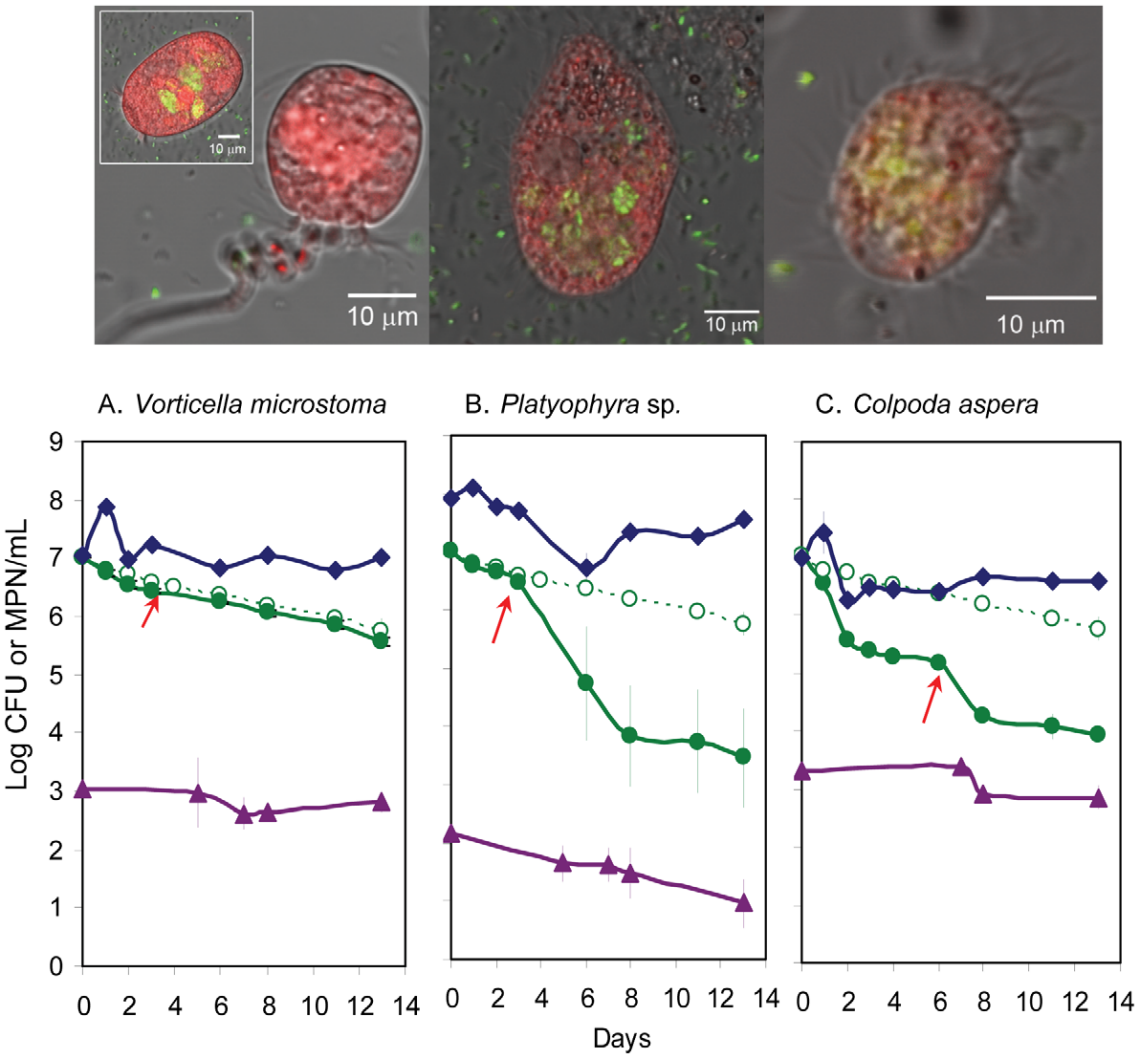
Predation of EcO157 by protozoa isolated from wastewater. The numbers of EcO157 strain MM123 (●, green), protozoa (▲, violet) and aerobic bacteria (♦, blue) in 0.01M PBS inoculated with GFP-EcO157 as the sole inoculated food source for *Vorticella microstoma* (A), *Platyophyra* sp (B) and *Colpoda aspera* (C) were monitored. PBS inoculated with only EcO157 (○, green) served as controls for the absence of predation. PBS was supplemented with 5% cereal grass medium (red arrows) after day 3 (A, B) or day 5 (C) to facilitate continued predation. Confocal images were obtained within 3 hours after inoculation of EcO157 and protozoa in PBS.

With GFP-EcO157 strain MM123 as the sole inoculated food source in PBS, *Platyophyra* and *Colpoda* consumed EcO157 cells in preference to the aerobic bacteria (Figure 5B and 5C) carried-over from wastewater. Addition of 50 µg/mL tetracycline to CGM used to prepare the protozoan inoculum did not eliminate any aerobic bacteria.

Although protozoan numbers maintained steadily throughout the incubations, *Vorticella* did not consume any EcO157 even after supplementation of 5% CGM (Figure 5A). However, supplementation of the *Platyophyra* and *Colpoda* samples resulted in significant consumption of EcO157 cells (Figures 5B and C). An initial 1.3-log decline of EcO157 cells was observed with *Colpoda*, followed by an additional smaller decline, but supplementation of 5% CGM on day 6 resulted in a further approximately 1-log decline of EcO157 at day 14 (Figure 5C). The number of *Colpoda* declined slightly also along with the decline of EcO157 populations after 8 days.

Although all three protozoa consumed GFP-EcO157 cells (Figure 5A to 5C), within 3 hours after inoculation of both EcO157 and protozoa to PBS, the majority of *Vorticella*, which appeared to be cysts with contractile stalks, did not ingest any GFP-EcO157 cells. Decreasing motility was observed during the 13-day incubation period, however, all motile vorticellids (Figure 5A, inset) consumed EcO157. Cross-sectional scans obtained for all three protozoa experiments confirmed that GFP-EcO157 were internalized (Figure 5).

Aerobic bacterial numbers remained relatively stable throughout the incubation of EcO157 with *Vorticella* in PBS, but appeared to be consumed initially along with EcO157 cells by *Colpoda*. In contrast, *Platyophyra* began to consume more aerobic bacteria and EcO157 after a supplementation of CGM at day 3 followed by an increase in the aerobic bacteria after 6 days and a significant decrease in EcO157 (Figure 5B).

### Nutrient addition to stimulate grazing of EcO157 by native protozoa

**Figure 6:**
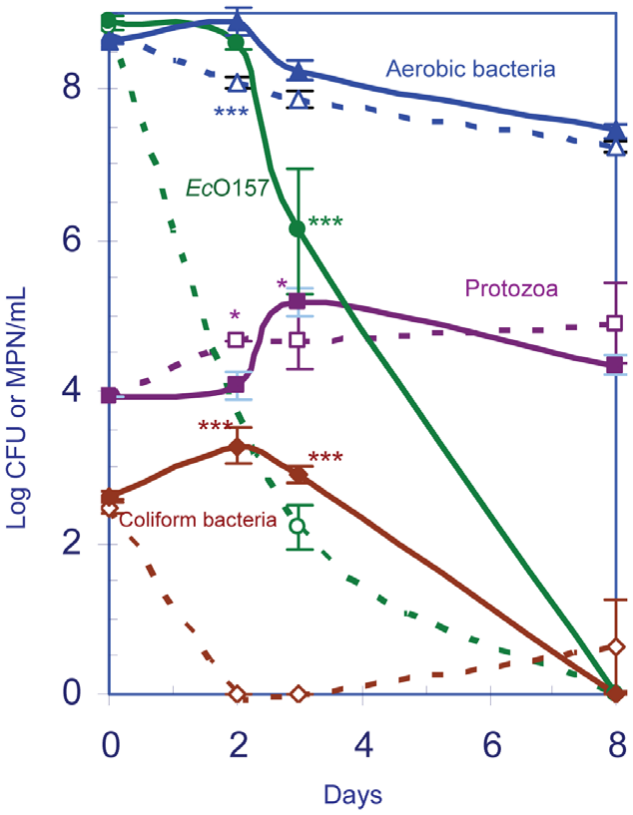
Addition of 10% LB to stimulate grazing of EcO157 by native protozoa. Fate of EcO157 strain MM149 (○, ●; green), native coliforms (◇, ♦; red), aerobic bacteria (△, ▲; blue), and protozoa (□, ■; violet) in wastewater microcosms supplemented with (solid lines and symbols) or without (broken lines and open symbols) 10% LB. Since EcO157 interferes with counting of coliform bacteria, coliform counts were obtained from uninoculated wastewater supplemented with (♦) or without (◇) 10% LB. Repeated-measures Two-way ANOVA indicated significant increases in protozoa (*P* = 0.0014) with 10% LB in wastewater inoculated with MM149. Source of variation with time was *P*≤0.0001 for aerobic bacteria, MM149, coliforms and protozoa. Bonferroni post hoc tests: *** = *P*<0.001; * = *P*<0.05.

Protozoa isolated from wastewater required supplemental nutrients to aid in the consumption of EcO157 strain MM123 (Figure 5). Similarly, supplementing wastewater with 10% LB broth (Figure 6) also increased significantly the growth of EcO157 strain MM149 and coliform bacteria after 2 days (*P*<0.001). Addition of LB resulted also in a 3-fold increase in protozoan numbers (1.6×10^5^ with LB vs 5×10^4^ with out) after a 3-day incubation (*P*<0.05). However, there was approximately a 4-log and 2-log higher decrease in EcO157 and coliforms at day 3, respectively, compared to the experiments with LB, indicating a corresponding increased fitness in *E. coli* and coliform bacteria in an environment with adequate nutrients (Figure 6). No increase in protozoan growth was observed at a low inoculum of 7×10^6^ CFU/mL of EcO157. However, in both instances of supplementation of LB broth in the presence or absence of high inocula, the protozoan numbers increased significantly within 2 to 3 days, then declined to background levels corresponding to native aerobic bacterial counts. Both EcO157 and native coliform bacteria declined to undetectable levels after 8 days regardless of the presence or absence of LB. The spike in native aerobic bacteria after 2 days resulted from enumerating the increased growth of EcO157 as aerobic bacteria (Figure 6).

## Discussion

We have shown conclusively that protozoa in a dairy lagoon wastewater microcosm graze and grow on EcO157 and decrease significantly populations of EcO157. The protozoa that consumed EcO157 in wastewater were isolated for further characterization of their identities and individual activities with EcO157.

We observed by confocal microscopy a rapid intake of GFP-EcO157 within 2 h by protozoa, and the subsequent release of food vacuoles filled with live cells of EcO157 within 3 days after ingestion (Figure 2). This result is similar to a previous report of the expulsion of food vacuoles containing EcO157 within 24 h after initiation of feeding by *Glaucoma* sp. isolated from store-bought lettuce [10]. Furthermore, we observed internalized GFP-EcO157 cells in some protozoa after 7 days, whereas GFP-EcO157 cells were absent from wastewater within 5 days after inoculation (Figure 1). This differs from an earlier finding of growth of EcO157 during 35 days in the presence of axenically grown *A. polyphaga* or its lysates [18]. Differences in results with different protozoa, pathogen strains and conditions emphasize the importance of further work to determine whether protozoa are a serious reservoir for some pathogens and can protect them from destruction by environmental stresses and, especially, whether hypervirulent pathogen phenotypes are selected and/or they replicate inside protozoa and their vacuoles for subsequent ejection into the agricultural environment.

We demonstrated incremental growth of native protozoa with sufficient populations of EcO157. This would be consistent with our previous study with *E. coli* ONT:H32 (MM158) inoculated four times sequentially into wastewater from dairy lagoons over a 45-day period [6]. In the current study, predation of EcO157 (MM123) was correlated with the incremental growth of protozoa above the level noted for growth obtained on native bacteria alone (Figure 1). Furthermore, an 11-fold increase in protozoan numbers was observed after they had consumed 99.9% of the second inoculum of 2×10^7^ EcO157 CFU/mL. Rates of consumption of bacteria by protozoa of 9 to 266 per hour for a flagellate and 200 to 5,000 per hour for a ciliate have been reported previously [29]. This corresponds to a protozoan population of 10^4^ consuming from 10^6^ to 10^9^ bacteria per day and, therefore, is consistent with protozoan increases after the second inoculation due to acclimation of protozoa to the EcO157 strain tested in this study. These results are in agreement with a recent observation of increased growth of protozoa in co-culture with EcO157, *S. enterica* and *Listeria monocytogenes* [10]. Since the protozoan numbers increased after only one re-inoculation in this study, we speculate that the significant decrease of 90% of *E. coli* ONT:H32 cells from 9.7 to 0.9 days after the 4^th^ re-inoculation [6] resulted from enhanced consumption of *E. coli* by specific protozoa [30]. This suggests adaptation of the protozoa to a specific bacterial strain present in high numbers compared to other natural strains present in the wastewater. Potential adaptation of predator biology of protozoa to bacteria prevalent in an environment, especially bacterial pathogens, would be an intriguing area for further study.

To distinguish protozoan predation from other biotic and abiotic factors, we confirmed that protozoa enriched from wastewater and grown in CGWM were responsible for a significant decrease in the population of outbreak strain of EcO157 (MM123). Furthermore, EcO157 grew in the absence of protozoa and maintained at ∼8 log CFU/mL. Direct evidence for predation of EcO157 in wastewater has not been reported, however, extended survival has been reported for EcO157 in filtered well water [22], in autoclaved wastewater from dairy lagoons [23] and cycloheximide-treated cattle trough water [31].

Protozoa enriched from wastewater consumed EcO157 preferentially (Figure 4) in comparison to the background aerobic bacterial populations in CGWM. Native bacteria were consumed as alternate prey when the preferred food source, EcO157, declined to a threshold level [32] of 10^5^ cells/mL after a 3-day incubation. If the predation is merely density dependent [33], aerobic bacteria would have been preferentially consumed during the first 3 days. Although protozoa are known to consume gram negative bacteria preferentially over gram positives [34], [35], this is the first report that protozoa from dairy lagoons consume EcO157 preferentially over natural microflora.

We have isolated and identified *V. microstoma*, *Platyophyra* sp. and *C. aspera* by culturing serial dilutions of the protozoan enrichments in CGWM. Apparently these protozoa in wastewater from dairy lagoons did not originate from the ruminants [36], [37], as suspected. It is likely that mixing of enrichments on a shaker might have eliminated the anaerobic ciliates *Entodinium* and *Epidinium* that are most prevalent in the cow rumen [37]. Distinct feeding patterns were detected for each protozoan in consuming EcO157 in PBS (Figure 5). Initially, *C. aspera* consumed simultaneously both EcO157 and aerobic bacteria carried over from wastewater, in contrast to *Platyophyra sp.* requiring a supplemental addition of 5% CGM as nutrients to initiate consumption of EcO157 and aerobic bacteria. However, *V. microstoma*, a common inhabitant of activated sludge systems [38], ingested (Figure 5A, inset), but failed to digest EcO157 during the 13 days of incubation. Neither the cysts of *Vorticella* (Figure 5A) nor any other cysts observed in wastewater samples contained GFP-EcO157. We detected motile vorticellids with vesicles filled with GFP-EcO157 after 7 days in wastewater (Figure 2) that was devoid of free EcO157 cells. It is possible that Shiga toxins [17] provided a selective advantage in resisting the digestion by *Vorticella*, in contrast to *Platyophyra* and *C. aspera* unaffected by Shiga toxin in consumption of EcO157. However, both *Platyophyra* and *Colpoda* failed to eliminate EcO157, possibly due to the exhaustion of supplemental nutrients required to stimulate grazing or for reaching a threshold level for consumption by protozoan cells, as observed for the efficient consumption of non-pathogenic *E. coli* by *T. pyriformis* [32].

The ability of EcO157 to be ingested and survive for 13-days in co-culture with *Vorticella*, *Platyophyra* and *Colpoda* support an earlier report of a protective role for protozoa during the transport of pathogenic bacteria to environments conducive for re-growth [11]. Indeed, a recent study reported that EcO157 released from expelled food vacuoles grew in spinach extracts [10] that represent potential nutrients present on leaf surfaces as a result of tissue damage or cut edges during or prior to harvest of produce [39]. Such a role for protozoa in sequestering bacterial pathogens in vacuoles resulting in enhanced survival has been reported also for *S. enterica* [10], [11], *S. typhimurium* [40], *M. avium* [15], *L. pneumophila* [41], [42], *Staphylococcus aureus* (MRSA) [43] and others [44]. If coexistence of pathogenic *E. coli* and predatory protozoa as observed in nutrient and alternate prey rich wastewater supplemented with CGM occurs in nature, the protozoa could be an effective refuge for bacteria in general, and foodborne pathogens in particular, thus serving as an environmental reservoir, as has been reported for coexisting *M. avium* and *A. polyphaga* [45]. Since motile cells of *V. microstoma* ingested EcO157 without digesting them (Figure 5A), they are candidates for “storing” and/or transporting pathogenic EcO157 in dairy environments.

In contrast to the failure of protozoan grazing at a certain critical density of prey discussed above, populations of EcO157 (Figures 1 and 6) inoculated into wastewater directly were eliminated without a noticeable threshold for the consumption of EcO157. This is predictable considering we observed 90% decline rates for several strains of EcO157 within 1 to 10 days in wastewater from different dairy lagoons in microcosms; EcO157 cells were eliminated in less than 4 weeks [6]. Since protozoa isolated from wastewater failed to eliminate EcO157 (Figures 4 and 5), we suspect other components of wastewater may be responsible for decreases, such as lytic phages [46], bdellovibrios [47], [48] and inhibitory chemicals [23], emphasizing the complex microbial ecology that may affect bacterial pathogens in the environment and food production systems.

Since supplemental nutrients aid in decreasing the populations of EcO157 by *Platyophyra* and *Colpoda* (Figure 5) and other protozoa in wastewater (Figure 6), attempts to augment beneficial protozoa in dairy lagoons could be a novel approach for on-farm control of zoonotic pathogens in wastewater before the water is distributed to other locations (feed crops, orchards, watersheds, etc.) or contaminates crops grown in proximity. Protozoa in feces and entering wastewater are affected by animal diet. High-grain diets [49], [50] often result in the decrease of protozoan cells and diversity of species. In addition, EcO157 declined rapidly in manure from cattle fed with 100% straw as compared with digestible silage supplemented with soy and maize concentrates [51]. Although, cattle diet might increase the beneficial protozoa that consume EcO157 in dairy lagoons, it could also result in stimulating protozoa that may protect and transport EcO157 to crops grown nearby to dairies.

To our knowledge, this is a first report of characterization of protozoa native to dairy lagoons that are capable of consuming but not eliminating the populations of EcO157. These protozoa are not reported to be common inhabitants of dairy animals, but they were capable of grazing on EcO157. Grazing of EcO157 by two of the three isolates of protozoa resulted in decreases in the populations of EcO157. Since all three protozoa failed to eliminate EcO157, but retained them in food vacuoles, a potential exists for EcO157 in protozoan vacuoles to re-colonize cattle in free-stall dairies that use recycled water commonly for washing lanes. This would result in a complex ecology and possible evolution of EcO157 or other zoonotic pathogens in protozoa in different wastewater conditions (nutrients, fecal load) and different cows. This complex ecology could involve specific interactions between protozoa and pathogens, elimination or protection of pathogens in protozoa, and protozoa adaptations specific to predominant bacteria in the agricultural environment. Further studies will be required to determine the reproducibility of these complex interactions, the detection of other factors and, potentially, the identification of novel interventions beneficial for food safety.

Our study provides new information about the risks associated with run-off from dairies and ranches [8] that could contaminate potentially nearby crops and orchards and possibly yield commodities that could become a major public health issue.

## Supporting Information

Method S1 Characterization of monocultures of protozoa by 18S rRNA sequencing
(DOC)

Figure S1 Confocal image of a protozoan taken 2 h after treating the wastewater with 1×10^5^ CFU of GFP-EcO157/mL. The compiled image was obtained with transmitted light, propidium iodide and GFP. Protozoa did not contain any internalized GFP-EcO157 cells.
(TIF)

Figure S2 Image of a flagellate taken 4 days after inoculating the wastewater with 9×10^7^ CFU of GFP-EcO157/mL. The flagellate ingested and retained live cells of EcO157 within food vacuoles although the surrounding wastewater contained little or no GFP-EcO157.
(TIF)

Figure S3 A protozoan with GFP-labeled cells attached to the surface. Confocal image was taken 2 h after inoculating the wastewater with 9×10^7^ CFU of GFP-EcO157/mL.
(TIF)
