# Supplementary material for: Identification of Protozoa in Dairy Lagoon Wastewater that Consume Escherichia coli O157:H7 Preferentially
Source: PLoS One. 2010 Dec 20;5(12):e15671. doi: 10.1371/journal.pone.0015671 (PMC3004959; doi:10.1371/journal.pone.0015671)
Supplement: Method S1 — Characterization of monocultures of protozoa by 18S rRNA sequencing (DOC) [file pone.0015671.s001.doc]

Method S1.

Characterization of monocultures of protozoa by 18S rRNA sequencing

Protozoa were grown in 100ml-portions of cereal grass medium, concentrated by centrifugation at 300 x g for 10 min and the cell pellet was used to extract template DNA used for the amplification of 18S rRNA sequences. Template DNA was obtained by disrupting the protozoan cells mixed with an equal amount of 0.1-mm silica-zirconium beads in a mini-bead beater (BioSpec Products, Bartlesville, OK). The tubes with protozoa-bead mixture were shaken five times of 60 sec pulses alternating with 60 sec cooling on ice. The cell debris was pelleted by centrifugation and the supernatant containing the DNA was used to amplify 18S rRNA as described by Karnati *et al.* [1] using the protozoa-specific forward primer P-SSU-342 (5’-CTTTCGATGGTAGTGTATTGGACTAC-3’) and a eukarya-specific reverse primer Medlin B (5’-TGATCCTTCTGCAGGTTCACCTAC-3’) with the following modification. Briefly, genomic DNA was amplified in ten 50-µl reaction mixtures containing 5 to 100 ng of template DNA, 500 nM of each primer and two Illustra PuReTaq Ready-To-Go PCR Beads (GE Healthcare, Piscataway, NJ). PCR reactions were carried out using ****DNA Engine Dyad® Peltier Thermal Cycler (Bio-Rad labs., Hercules, CA)**** using the following parameters: one cycle of 94^O^C for 2 min; 30 cycles of 94^O^C for 60 sec, 37^O^C for 65 sec, and 72^O^C for 3 min; and a final 6-min extension at 72^O^C. PCR products were purified with DNA Clean and Concentrator-5 kit (Zymo Research, Orange, CA) by using 5 volumes of DNA binding buffer to each volume of PCR product mixture as per manufacture’s instructions. PCR products were cloned using the TOPO TA cloning kit as per the manufacturer’s instructions and transformed into *E. coli* TOP10F’ One Shot competent cells (Invitrogen). Two PCR reactions were performed for each protozoan, and 5 clones were picked from each PCR to minimize potential PCR bias. DNA templates for sequencing were prepared using the Illustra Templiphi 100 ampliﬁcation kit (GE Healthcare) as described by the manufacturer. Sequencing reactions were performed as per the manufacturer’s instructions for the Big Dye Terminator v3.1 cycle sequencing kit (Applied Biosystems, Foster City, CA) using the Medlin B [1] reverse primer. Cycle sequencing reactions were purified using DyeEx 96 kit (Qiagen, Valencia, CA) and sequencing was carried out using an Applied Biosystems 3730 DNA analyzer. DNA sequences were trimmed at both the 5’ and 3’ ends to correct falsely called bases by using Lasergene SeqMan Pro (v7.0, DNASTAR, Inc., Madison, WI). Only sequences with unambiguous reads of >500 bp were used; each read used averaged approximately 600 bp. The compiled sequence data sets were aligned with the closest sequence relatives from the GeneBank database by using Kodon (v3.5, Applied Maths, Inc., Austin, TX).

**REFERENCE**

1. Karnati SK, Yu Z, Sylvester JT, Dehority BA, Morrison M, et al. (2003) Technical note: Specific PCR amplification of protozoal 18S rDNA sequences from DNA extracted from ruminal samples of cows. J Anim Sci 81: 812-815.
