# Supplementary figures and images for: Identification of Protozoa in Dairy Lagoon Wastewater that Consume Escherichia coli O157:H7 Preferentially
Source: PLoS One. 2010 Dec 20;5(12):e15671. doi: 10.1371/journal.pone.0015671 (PMC3004959; doi:10.1371/journal.pone.0015671)

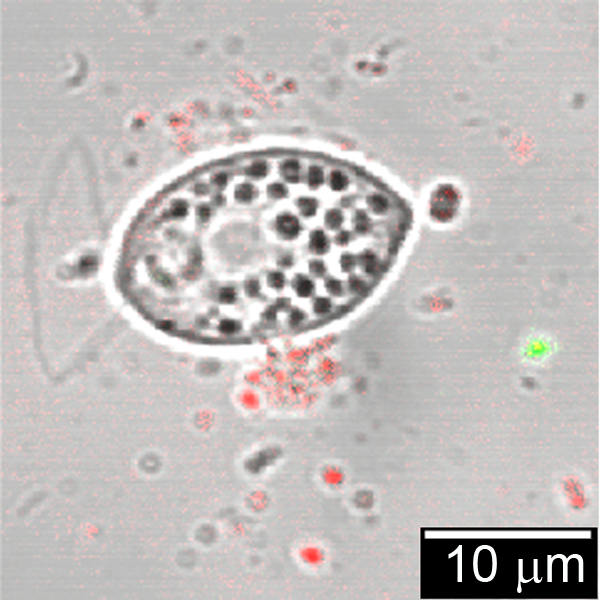

Supplement: Figure S1 — Confocal image of a protozoan taken 2 h after treating the wastewater with 1×10^5^ CFU of GFP-EcO157/mL. The compiled image was obtained with transmitted light, propidium iodide and GFP. Protozoa did not contain any internalized GFP-EcO157 cells. (TIF) [file pone.0015671.s002.tif]

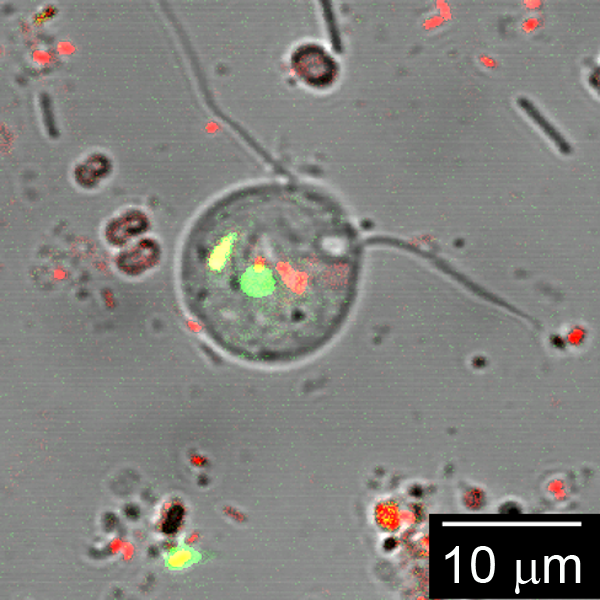

Supplement: Figure S2 — Image of a flagellate taken 4 days after inoculating the wastewater with 9×10^7^ CFU of GFP-EcO157/mL. The flagellate ingested and retained live cells of EcO157 within food vacuoles although the surrounding wastewater contained little or no GFP-EcO157. (TIF) [file pone.0015671.s003.tif]

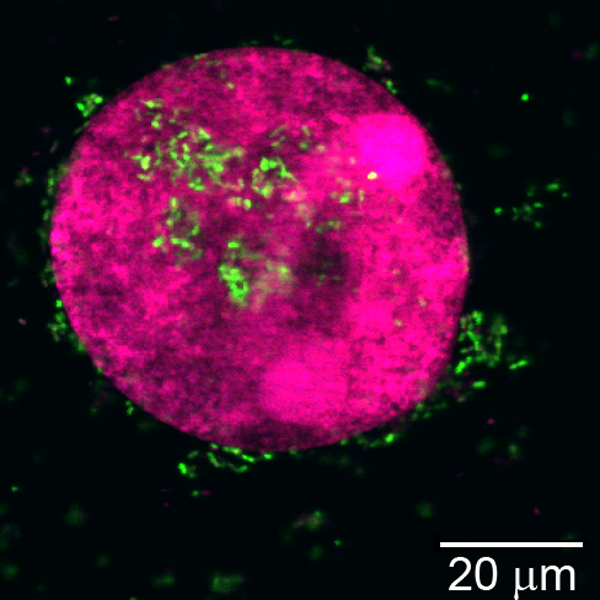

Supplement: Figure S3 — A protozoan with GFP-labeled cells attached to the surface. Confocal image was taken 2 h after inoculating the wastewater with 9×10^7^ CFU of GFP-EcO157/mL. (TIF) [file pone.0015671.s004.tif]
